# Multi-Target Approach for Drug Discovery against Schizophrenia

**DOI:** 10.3390/ijms19103105

**Published:** 2018-10-10

**Authors:** Magda Kondej, Piotr Stępnicki, Agnieszka A. Kaczor

**Affiliations:** 1 Department of Synthesis and Chemical Technology of Pharmaceutical Substances, Faculty of Pharmacy with Division of Medical Analytics, Medical University of Lublin, 4A Chodźki St., Lublin PL-20093, Poland; magda.kondej@onet.pl (M.K.); piotr.stepnicki93@gmail.com (P.S.); 2 School of Pharmacy, University of Eastern Finland, Yliopistonranta 1, P.O. Box 1627, Kuopio FI-70211, Finland

**Keywords:** antipsychotics, drug design, multi-target drugs, polypharmacology, schizophrenia

## Abstract

Polypharmacology is nowadays considered an increasingly crucial aspect in discovering new drugs as a number of original single-target drugs have been performing far behind expectations during the last ten years. In this scenario, multi-target drugs are a promising approach against polygenic diseases with complex pathomechanisms such as schizophrenia. Indeed, second generation or atypical antipsychotics target a number of aminergic G protein-coupled receptors (GPCRs) simultaneously. Novel strategies in drug design and discovery against schizophrenia focus on targets beyond the dopaminergic hypothesis of the disease and even beyond the monoamine GPCRs. In particular these approaches concern proteins involved in glutamatergic and cholinergic neurotransmission, challenging the concept of antipsychotic activity without dopamine D_2_ receptor involvement. Potentially interesting compounds include ligands interacting with glycine modulatory binding pocket on *N*-methyl-d-aspartate (NMDA) receptors, positive allosteric modulators of α-Amino-3-hydroxy-5-methyl-4-isoxazolepropionic acid (AMPA) receptors, positive allosteric modulators of metabotropic glutamatergic receptors, agonists and positive allosteric modulators of α7 nicotinic receptors, as well as muscarinic receptor agonists. In this review we discuss classical and novel drug targets for schizophrenia, cover benefits and limitations of current strategies to design multi-target drugs and show examples of multi-target ligands as antipsychotics, including marketed drugs, substances in clinical trials, and other investigational compounds.

## 1. Introduction

Schizophrenia is a severe mental illness, affecting up to 1% of the population, with major public health implications. The causes of schizophrenia might be genetic or environmental or both but the complex pathomechanism of this disease is not sufficiently understood. The clinical picture of schizophrenia involves three groups of symptoms, i.e., positive, such as hallucinations, delusions and other thought disorders, negative, including social withdrawal, apathy and anhedonia, and cognitive deficits like memory and learning impairments or attention deficiencies [1]. It is generally agreed that the symptoms of schizophrenia result from disturbances in neurotransmission involving a significant number of receptors and enzymes, mainly within the dopaminergic, glutamatergic, serotoninergic, and adrenergic systems. In this regard, the dopaminergic hypothesis is still the main concept of the disease and all marketed antipsychotics target dopamine D_2_ receptor. The dopaminergic hypothesis of schizophrenia evolved from the simple idea of excessive dopamine through the hypothesis combining prefrontal hypodopaminergia and striatal hyperdopaminergia and then to the current aberrant salience hypothesis [2]. However, novel findings in the field of neuroscience link schizophrenia with factors beyond the dopaminergic hypothesis and emphasize in particular the role of glutamatergic system in the development of the disease [3].

In order to treat efficiently complex neuropsychiatric diseases such as schizophrenia it is necessary to go beyond the “magic bullet” concept. This approach in drug discovery was based on the assumption that single-target drugs are safer as they have fewer side effects due to their selectivity. It turned out, however, that this is only true for single-gene diseases and the number of original single-target drugs were performing far behind expectations in the last ten years. Thus, “one-drug-one-target” paradigm has been gradually replaced by the concept of multi-target drugs (MTDs), sometimes termed “magic shotgun”. From the historical perspective, MTDs, in contrast to clean single-target drugs, were sometimes referred to as dirty or promiscuous drugs. In the case of diseases with complex pathomechanisms, such as neuropsychiatric diseases or cancer, single-targets medications have been demonstrated to a great extent a failure. Most potent antipsychotics, in particular second generation or atypical antipsychotics, target simultaneously a number of aminergic G protein-coupled receptors (GPCRs). Clozapine, which is used to treat drug-resistant schizophrenia, has nanomolar affinity to several aminergic GPCRs.

In this scenario drug design and discovery today has moved from the molecular and cellular level to the systems-biology-oriented level [4] to reflect subtle events occurring on the biological networks which lead to the disease [5]. Network pharmacology involves important aspects such as connectivity, redundancy and pleiotropy of biological networks [6] which clearly shows that most drug interact with more than one target. MTDs have a number of advantages over single-target drugs, including improved efficacy due to synergistic or additive effects, better distribution in the target tissue, accelerated therapeutic efficacy in terms of clinical onset and achievement of full effect, predictable pharmacokinetic profile and fewer drug-drug interactions, lower risk of toxicity, improved patient compliance and tolerance and lower risk of target-based drug resistance due to modulation of a few targets [7]. However, it is not easy to design potent MTDs and problems arise starting from a proper target selection through affinity balancing to avoiding affinity to related off-targets.

In this review we present classical and novel drug targets for the treatment of schizophrenia, discuss benefits and limitations of MTDs and their design, as well as present multi-target antipsychotics including marketed compounds, compounds in clinical studies, and other investigational compounds. The literature search for this review was mainly based on searching PubMed database with the search terms: schizophrenia, schizophrenia drug targets, antipsychotics, multi-target antipsychotics, multi-target ligands, multi-target drugs with the focus on the references from the last five years, in particular regarding novel investigational compounds.

## 2. Drug Targets for the Treatment of Schizophrenia

### 2.1. Dopamine and Serotonin Receptors

Most of currently available antipsychotic drugs (excluding third generation drugs) act by blocking dopamine receptors in central nervous system, as seen in Table 1. This is the classical way to treat schizophrenia. The original dopamine hypothesis of schizophrenia was proposed by Carlsson (awarded a Nobel Prize in 2000) on the basis of indirect pharmacological evidence in humans and experimental animals. In humans, amphetamine causes the release of dopamine in the brain and can produce a behavioral syndrome that resembles an acute schizophrenic episode. Hallucinations are also a side effect of levodopa and dopamine agonists used in Parkinson’s disease. In animals, dopamine release causes a specific pattern of stereotyped behavior that is reminiscent of the repetitive behaviors sometimes observed in patients suffering from schizophrenia. Potent D_2_ receptor agonists, such as bromocriptine, lead to similar effects in animals, and these drugs, like amphetamine, aggravate the symptoms of schizophrenic patients. Moreover, dopamine antagonists and drugs blocking neuronal dopamine storage (e.g., reserpine) are effective in controlling the positive symptoms of schizophrenia and in preventing amphetamine-induced behavioral changes [8].

It is now thought that positive symptoms are the result of overactivity in the mesolimbic dopaminergic pathway (the neuronal projection from the ventral tegmental area (VTA) to the nucleus accumbens, amygdala and hippocampus) activating D_2_ receptors, whereas negative symptoms may result from a lowered activity in the mesocortical dopaminergic pathway (the projection from the VTA to areas of the prefrontal cortex) where D_1_ receptors predominate. Other dopaminergic pathways in the central nervous system (i.e., nigrostriatal and tuberoinfundibular) seem to function normally in schizophrenia. Thus, in terms of treatment it would be desirable to inhibit dopaminergic transmission in the limbic system but enhance this transmission in the area of prefrontal cortex [9].

Besides antagonism to the dopamine D_2_ receptor, majority of antipsychotic drugs, especially those classified as second generation antipsychotics also block a wide range of other receptors, such as other dopamine receptors (D_1_, D_3_ or D_4_), serotonin (especially 5-HT_2A_ and 5-HT_2C_), histamine (especially H_1_) and α_1_-adrenergic. Interaction of antipsychotics with those receptors is associated mainly with occurrence of side effects, such as sedation and drowsiness (H_1_ receptors), weight gain (H_1_ and 5-HT_2C_), sexual dysfunction (5-HT_2_), or orthostatic hypotension (α_1_-adrenergic receptors). On the other hand, there are also hypotheses that antagonism to serotonin 5-HT_2A_ receptor may have beneficial effects when it comes to occurrence of extrapyramidal side effects, as well as to reducing negative and cognitive symptoms of schizophrenia. Basis of schizophrenia is still poorly understood and there are several hypotheses, which involve different neurotransmitters and receptors and try to explain their role in the pathogenesis of the disorder [12].

The serotonin hypothesis of schizophrenia is based on the studies of interactions between the hallucinogenic drug, LSD, and serotonin. Observations of the antipsychotic effects of drugs which are serotonin and dopamine antagonists (e.g., risperidone, clozapine) have resulted in the increased interest in serotonin receptors as a possible target for drugs used in the treatment of schizophrenia.

There are evidences that the efficacy and tolerability of the atypical antipsychotic drugs, such as clozapine, olanzapine, quetiapine, risperidone, and ziprasidone in the treatment of schizophrenia may result, in part, from their interaction with various serotonin receptors, in particular 5-HT_2A_ and 5-HT_1A_ receptors, what is the reason of growing interest in the role, which serotonin plays in the mechanism of action of antipsychotics. The antagonism to 5-HT_2A_ receptors, which is relatively potent, is connected with weaker antagonistic properties to dopamine D_2_ receptors and is the only common pharmacologic feature of atypical antipsychotic drugs. The subtypes of serotonin 5-HT receptors, that are involved in the pharmacological action of second generation antipsychotics, such as clozapine, or that may potentially serve as targets for better tolerated and more effective new antipsychotic agents, include: 5-HT_1A_, 5-HT_2A_, 5-HT_2C_, 5-HT_3_, 5-HT_6_, and 5-HT_7_ receptors [13].

The distribution of serotonin 5-HT_2A_ receptor in the central nervous system is wide, but the highest concentrations occur in the cortex. 5-HT_2A_ as well as 5-HT_1A_ receptors are located on the neurons that play significant role in schizophrenia. Those are cortical and hippocampal pyramidal glutamatergic neurons and γ-aminobutyric acid (GABA) interneurons. Serotonin 5-HT_2A_ receptors localized on GABAergic interneurons stimulate the release of γ-aminobutyric acid and in that way play an important role in the regulation of the neuronal inhibition. 5-HT_2A_ receptors are distributed also in the substantia nigra and ventral tegmentum from which arise the nigrostriatal and mesocorticolimbic dopaminergic neurons. 5-HT_2A_ receptors modulate the activity of dopaminergic neurons. Antipsychotics that act by blocking serotonin 5-HT_2A_ receptor (e.g., clozapine, risperidone) lead to the increased release of dopamine in the striatum by decreasing the inhibitory effect of serotonin, what manifests clinically in reducing extrapyramidal effects. It is also suggested that combined effects of antagonism at dopamine D_2_ and serotonin 5-HT_2A_ receptors in the mesolimbic circuit counteract the excessive dopamine transmission, which leads to occurrence of positive symptoms of schizophrenia. Moreover, improvement of the negative symptoms is associated with antagonism at 5-HT_2A_ receptor, due to enhanced release of both dopamine and glutamate in the mesocortical pathway [9,13].

The behavioral evidence of interactions between serotonin 5-HT_2A_ receptor and dopamine rests on the effect of 5-HT_2A_ receptor antagonists on locomotor activity stimulated by amphetamine. Namely, giving low doses of amphetamine to rodents results in producing in them locomotor hyperactivity, which is mediated by the release of dopamine from the dopaminergic neurons in the mesolimbic circuit. This amphetamine stimulated hyperactivity is observed to be inhibited by first and second generation antipsychotic drugs and is thought to be an effect of antagonism to dopamine D_2_ receptor, which all of those drugs share as a mechanism of action. However, some observations proved that compounds, such as amperozide, which are antagonists selective to serotonin 5-HT_2A_ receptor and do not exhibit any affinity for dopamine D_2_ receptor, also lead to lowering of hyperactivity in mice stimulated by administration of amphetamine [14]. These results support the concept that compounds that are antagonists to 5-HT_2A_ receptor may improve behavioral states associated with excessive activity of dopaminergic neurons and may serve as effective antipsychotic medications.

Typical antipsychotic drugs, beside blocking dopamine D_2_ receptors in the mesolimbic circuit, act also antagonistic to D_2_ receptors localized in the nigrostriatal pathway, what is thought to result in occurrence of extrapyramidal side effects. Low doses of amphetamine administered to rodents lead to producing exploratory locomotor activity, whereas high doses of amphetamine causes the occurrence of repetitive, stereotyped behaviors, which are similar to those produced by the direct agonist of dopamine D_2_ receptor, apomorphine. Those stereotyped behaviors are inhibited by first generation antipsychotics, what suggests that their antagonist properties are the cause of producing extrapyramidal side effects. Contrarily, amperozide and other antagonists of the serotonin 5-HT_2A_ receptor do not reduce repetitive behaviors induced by apomorphine or high doses of amphetamine. These findings suggest that antipsychotic drugs which are antagonists to 5-HT_2A_ receptor do not cause extrapyramidal side effects, in contrast to first generation drugs, which are devoid of activity to serotonin receptors.

The majority of clinical studies of serotonin 5-HT_2A_ receptor antagonists have been carried out using ritanserin, the compound that exhibits antagonist properties to both 5-HT_2A_ and 5-HT_2C_ receptors. Its effectiveness has been studied in monotherapy, as well as an adjunct to existing treatment with antipsychotics. The studies have led to conclusions that ritanserin improves in particular negative symptoms of schizophrenia, which were poorly ameliorated in case of treatment with typical antipsychotic drugs [15].

To sum up, due to ability of antagonists of serotonin 5-HT_2A_ receptor to interfere with elevated activity of dopamine, the antagonism of this receptor is believed to contribute to improvement of both positive and negative symptoms of schizophrenia and to causing less extrapyramidal side effects than older antipsychotics [16].

The 5-HT_1A_ receptor is the subtype of serotonin receptors that is probably the best characterized in terms of functioning. It plays a significant role in modulating the activity of monoaminergic, inter alia dopaminergic, neurons. The functioning of 5-HT_1A_ receptor may be described as antagonistic to the serotonin 5-HT_2A_ receptor, when it comes to both presynaptic and postsynaptic its localization. Activation of serotonin 5-HT_1A_ inhibitory autoreceptors located in the cells of raphe nucleus leads to inhibition of those neurons. In contrast, 5-HT_2A_ receptors while activated in general cause the activation of serotonergic neurons by several mechanisms, which include a direct or indirect inhibition of GABAergic inhibitory interneurons, and a direct mechanism of excitation of other neurons, inter alia glutamatergic neurons. Both postsynaptical 5-HT_1A_ and 5-HT_2A_ receptors are located in the cortex on the pyramidal neurons. Activation of this 5-HT_1A_ receptor results in neuronal inhibition through activation of potassium current, what leads to hyperpolarization. Contrary, 5-HT_2A_ receptor while activated, facilitates neuronal output in the mechanism of activation of phospholipase C. Serotonin 5-HT_1A_ receptors are suggested to be localized also presynaptically on GABA neurons terminals and pre- or postsynaptically on the GABAergic interneurons in the dentate gyrus in the hippocampus. Basing on the opposition between those two serotonin receptors, it is thought that agents acting as 5-HT_1A_ receptor agonists are able to modulate dopaminergic transmission in the central nervous system in a similar way to antagonists to serotonin 5-HT_2A_ receptor. Agonists to 5-HT_1A_ receptor may both induce the dopamine release in the prefrontal cortex and potentiate the inhibiting effect on dopamine release of dopamine D_2_ receptor antagonists [17].

In the brains of patients suffering from chronic schizophrenia, the density of serotonin 5-HT_1A_ receptors is increased, what suggests a close correlation between pathogenesis of the disease and serotonin 5-HT_1A_ receptors. These receptors are now considered as preferable target to treat schizophrenia, since there are evidences that stimulation of serotonin 5-HT_1A_ receptors may contribute to decreasing of extrapyramidal side effects induced by antipsychotics [18] and ameliorating affective disorders such as depression or anxiety [19]. Moreover, blockade of 5-HT_1A_ receptors may result in improvement of cognitive symptoms of schizophrenia [20].

It has been proved in different studies that agents which are selective agonists of serotonin 5-HT_1A_ receptor, such as tandospirone or buspirone, reduced extrapyramidal side effects (e.g., bradykinesia, catalepsy) induced by antipsychotics from first generation [21]. Agonists of 5-HT_1A_ receptor are thought to reduce extrapyramidal side effects induced by neuroleptics in the way of stimulating serotonin 5-HT_1A_ receptors localized postsynaptically, since the inactivation of serotonergic neurons by p-chlorophenylalanine had no impact on the actions of 5-HT_1A_ receptor agonists, when it comes to alleviating extrapyramidal side effects [22].

Reducing cognitive symptoms of schizophrenia is another significant role of serotonin 5-HT_1A_ receptors. Cognitive dysfunction belongs to those symptoms of schizophrenia, whose treating with currently available drugs is still not very effective. Some of recently carried clinical studies have proved that the partial agonist properties of tandospirone regarding 5-HT_1A_ receptor relevantly improved the deficits in cognition in schizophrenic patients. Studies carried on animals also showed that 5-HT_1A_ receptor antagonists improved the cognitive deficits induced by antagonists to mACh receptor, such as scopolamine, or antagonists of *N*-methyl-d-aspartate (NMDA) receptor [23]. Although further studies are required, there are findings which suggest that serotonin 5-HT_1A_ receptor antagonists may contribute to managing schizophrenia on account of ameliorating cognitive impairments [24].

Many compounds that bind to serotonin 5-HT_2A_ receptors also exhibit an affinity to the structurally related serotonin 5-HT_2C_ receptor. There are evidences that support the idea of an antipsychotic potential for antagonists of 5-HT_2C_ receptor. One of them concerns meta-chlorophenylpiperazine (mCPP), which act as an agonist of serotonin 5-HT_2C_ receptor [25]. The main action of mCPP in humans may be described as a selective activation of serotonin 5-HT_2C_ receptors [26]. mCPP causes the worsening of positive symptoms in schizophrenic patients but pretreatment with mesulergine, which is an antagonist to 5-HT_2_ receptor, results in decreased level of psychotic episodes, induced by the drug [27]. It is suspected that 5-HT_2C_ receptor antagonists inhibit dopaminergic activity in mesolimbic and nigrostriatal pathways and thus contribute to reducing symptoms of schizophrenia and alleviating extrapyramidal side effects. Nonetheless, the role of this subtype of serotonin receptor in the pathogenesis of schizophrenia is still poorly understood and requires further studies [28].

Although dopamine and serotonin receptors are classical drug targets for the treatment of schizophrenia, novel drugs acting through these receptors can be developed based on novel signaling mechanisms typical for the family of GPCRs. These include allosteric modulators [29], biased ligands [30], compounds acting on receptor dimers, oligomers and mosaics [31,32,33,34] and last but not least intentionally promiscuous multi-target ligands [35].

### 2.2. Adrenergic and Histaminergic Receptors

Noradrenaline has a key role in the pathomechanism of schizophrenia although the specific role of α adrenergic receptors has been not well elucidated yet [36]. It has been hypothesized that interactions of atypical antipsychotics with α-adrenergic receptors contributes to their atypicality [37]. It was shown that antagonism at α_1_ adrenergic receptors is beneficial to treat positive symptoms, in particular in acute schizophrenia while antagonism at α_2_ adrenergic receptor, characteristic for clozapine and to some extent risperidone might be important to relieve negative symptoms and cognitive impairments [37]. Blockade of α adrenergic receptors may have a stabilizing effect on the dopaminergic neurotransmission in schizophrenia. In contrast, it was also reported that activation of α_2A_ adrenergic receptors in prefrontal cortex may improve cognitive functions [38]. Moreover, adjunctive α_2_ adrenergic receptors antagonism increases the antipsychotic activity of risperidone and promotes cortical dopaminergic and glutamatergic, NMDA receptor-mediated neurotransmission [39]. It was also shown that blockade of α_2C_ adrenergic receptors alone or in combination with dopamine D_2_ receptor blockade could be also beneficial in schizophrenia [38].

The histamine H_1_ receptor is a classical off-target for antipsychotics as its blockade causes sedation and may be involved in weight gain. Although weight gain and metabolic disorders can also be attributed to blockade of adrenergic or cholinergic receptors, antagonism of histamine H_1_ receptors is described as a key reason for second generation antipsychotics-induced obesity [40]. In contrast, the histamine H_3_ receptor is an emerging target for novel antipsychotics [41] as selective antagonists or inverse agonists of this histamine receptor subtype are efficient in treatment cognitive deficiencies in schizophrenia [42].

### 2.3. Muscarinic and Nicotinic Receptors

Muscarinic receptors have a pivotal role in modulating synaptic plasticity in the prefrontal cortex and stimulation of these receptors results in long-term depression at the hippocampo-prefrontal cortex synapse [43]. A growing body of evidence indicates central role of disturbances in cholinergic neurotransmission in schizophrenia [44]. Postmortem studies indicate a reduced number of cholinergic interneurons in the ventral striatum in schizophrenia patients [45]. Furthermore, neuroimaging studies indicated that muscarinic receptors availability was significantly less in schizophrenia patients and positive symptoms of schizophrenia are negatively correlated with muscarinic receptors availability [46]. It should be emphasized that muscarinic receptor antagonists worsen cognitive and negative symptoms in schizophrenia patients and xanomeline, a muscarinic receptor agonist, ameliorates all symptoms in schizophrenia patients and corresponding animal models [43]. Based on these and other findings muscarinic hypothesis of schizophrenia has been suggested [47].

Involvement of nicotinic cholinergic receptors in the pathomechanism of schizophrenia can explain why schizophrenia patients are often heavy smokers [48,49]. It is assumed that smoking relieves particularly negative symptoms of schizophrenia. More and more evidence indicates that activation of α_7_ nicotinic receptors [50] by agonists or positive allosteric modulators can be a promising strategy for the treatment of schizophrenia [51,52].

### 2.4. Metabotropic and Ionotropic Glutamatergic Receptors

Glutamate is one of the main excitatory neurotransmitters in the mammalian central nervous system [53]. Glutamatergic pathways linking to the cortex, the limbic system, and the thalamus regions are crucial in schizophrenia [54,55]. Abnormalities in the glutamatergic neurotransmission may influence synaptic plasticity and cortical microcircuitry, particularly NMDA receptor functioning [56]. NMDA receptors are ligand-gated ion channels, and are pivotal for excitatory neurotransmission, excitotoxicity and plasticity [57,58].

Glutamatergic hypothesis of schizophrenia is based on the observation that antagonists of *N*-methyl-d-aspartate (NMDA) receptors, such as phencyclidine or ketamine produce schizophrenia-like positive, negative, and cognitive symptoms in animal models and healthy individuals [59,60]. Glutamatergic hypothesis of schizophrenia is mainly a concept of hypofunction of NMDA receptors in this disease, however other ionotropic glutamate receptors (α-amino-3-hydroxy-5-methyl-4-isoazolepropionic acid, AMPA and kainate receptors) as well as metabotropic glutamate receptors are also involved.

In therapeutic trials compounds which promote NMDA receptor signaling were found relieve certain symptoms in patients with schizophrenia [61]. Moreover, in postmortem studies abnormalities in glutamatergic receptor density and subunit composition in the prefrontal cortex, thalamus, and temporal lobe were reported [62,63,64], and these are brain parts with altered stimulation during cognitive actions performed by schizophrenia patients [65]. NMDA receptor hypofunction may result in morphological and structural brain changes leading to the onset of psychosis [66,67]. It was suggested that levels of glutamate decrease with age in healthy people, but it was not found if they are influenced in case of chronic schizophrenia [68].

Antipsychotics may interfere with glutamatergic neurotransmission by influencing the release of glutamate, by modulation glutamatergic receptors, or by changing the density or subunit composition of glutamatergic receptors [55]. It was shown that antipsychotics blocking dopamine D_2_ receptor increase the phosphorylation of the NR1 subunit of the NMDA receptor, thus promote its activation and consequent gene expression [69]. In this regard dopamine–glutamate interactions occur intraneuronally and intrasynaptically. There are also findings that certain second generation antipsychotics act on NMDA receptors in a distinct way than the first generation antipsychotics [70].

Abnormalities in glutamatergic neurotransmission constitute a possible drug target for schizophrenia, in particular for the treatment of cognitive impairment and negative symptoms [54,55]. Reports about hypoactivity of NMDA receptors in schizophrenia led to clinical trials with ligands stimulating this receptor [55]. Classical NMDA receptor agonists are not considered here due to excitotoxicity and neuron damage resulting from excessive NMDA receptor stimulation. In this regard, the glycine modulatory binding pocket on the NMDA receptor might be an attractive drug target [71]. Next, positive allosteric modulators of AMPA receptors [72,73] as well as orthosteric ligands and modulators of metabotropic glutamatergic receptors [74], in particular ligands acting on mGluR2/3 receptors [75] might be considered promising potential medications against schizophrenia in agreement with the glutamatergic hypothesis of this disease.

### 2.5. Other Drug Targets in Schizophrenia

There are also potential drug targets for the treatment of schizophrenia beyond transmembrane receptors. Most important enzymes with implications in schizophrenia include the serine/threonine kinase glycogen synthase kinase-3 (GSK-3) involved in cognitive-related processes such as neurogenesis, synaptic plasticity and neural cell survival [76], cyclic nucleotide (cNT) phosphodiestereases (PDEs)-intracellular enzymes which governs the activity of key second messenger signaling pathways in the brain [77] and acetylcholinesterase for treatment of cognitive impairments [78].

## 3. Multi-Target Compounds: Strategies of Design, Benefits, and Limitations

As has already been mentioned, during last twenty years most efforts in drug design and discovery followed the paradigm “one disease, one gene, one molecular target, one drug”. However, novel findings in the field of systems biology and discoveries of molecular complexity of illnesses considerably moved current drug discovery efforts towards multi-target drugs [79,80]. Such compounds are able to exert numerous pharmacological actions and have emerged as magic shotguns in the treatment of multifactorial diseases in contrast to classical magic bullet approach [81].

### 3.1. Design of Multi-Target Compounds

**Figure 1:**
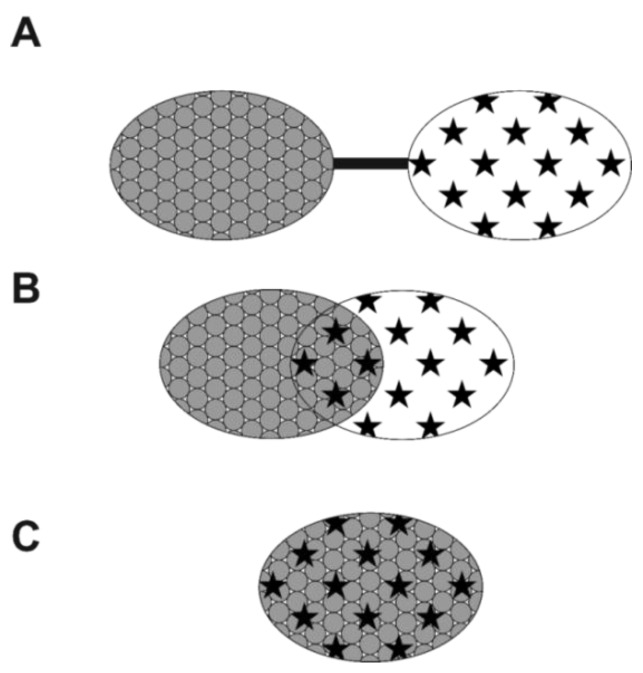
Strategies to design multi-target ligands. (**A**) linking of pharmacophores; (**B**) overlapping of pharmacophores; (**C**) integration of pharmacophores.

Classical approaches to design multi-target ligands involve three different ways of combination of two pharmacophores, leading to a cleavable conjugate where two pharmacophores are connected by a linker (a modern form of combination therapy), a compound with overlapping pharmacophores or a highly integrated multi-target drug, as seen in Figure 1 [5]. Multi-target drugs, in particular those obtained by pharmacophore integration strategy are referred to as “master key compounds” [82,83]. Thus, MTDs are designed broadly as hybrid or conjugated drugs or as chimeric drugs from two or more pharmacophores/drugs having specific pharmacological activities [84].

Morphy and Rankovic [85] described two approaches for designing multi-target drugs: knowledge-based strategies and screening strategies. Knowledge-based techniques are based on available biological data from old drugs or other bioactive compounds, from either literature or proprietary company sources. Other methods include the screening of either diverse or focused compound libraries. Classical diversity based screening is the high-throughput screening (HTS) of large and differentiated compound collections versus one protein, and hits found are then triaged on the basis of activity at the other protein. In focused screening, compounds known to have robust activity at one protein are screened for activity at the other one. Even if only moderate activity is found for the second protein, it can supply a useful baseline for increasing that activity by incorporating structural elements from more potent selective ligands for this target [85].

Modern in silico approaches can be also used to design multi-target ligands and can be classified into ligand-based and structure-based strategies [4]. Ligand-based target fishing strategies rely either on similarity-based screening or machine learning methods [4]. Moreover, ligand-based pharmacophores can be used. The advantage of this approach is independence from available structural information on the protein. These methods involve 2D or 3D similarity searches. Polyphramacological profiling of the compounds may also be based on three-dimensional structure-activity relationship (3D-QSAR) techniques [7]. Structure-based methods involve molecular docking (e.g., docking-based virtual screening [86] and inverse docking) or structure-based pharmacophores. The advantage of structure-based approaches in comparison to ligand-based approaches is that they do not rely on available activity data [4].

The main principle in designing multi-target compounds is the achievement of superior therapeutic efficacy and safety by targeting multiple players in pathogenic cascade simultaneously [4].

### 3.2. Advantages and Disadvantages of Multi-Target Ligands

Multifunctional ligands are particularly interesting as their molecules have common parts responsible for activity, and their structure is formed as a result use of pharmacophore fragments. Receiving such hybrid compounds allows not only to improve their activity, but also to positively affect pharmacokinetic parameters, similar to those shown by drugs used in therapy [81].

The main advantages of multi-target-drugs compared to single-target drugs and combination therapy include: (i) reflecting the complex pathomechanism of the disease and better therapeutic efficacy and (ii) better therapeutic safety avoidance of different bioavailabilities, pharmacokinetics, and metabolism of a combination regimen and avoidance of drug–drug interactions [87]. Multi-target mode of action is beneficial to combat drug resistance and development of tolerance and can be also a base of drug repurposing. The disadvantage of MTDs is the difficulty in designing compounds with balanced activity to multiple targets, sometimes resulting in a need to compromise activity at some targets. Moreover, compounds obtained in particular by pharmacophore linkage are often not drug-like due to high molecular mass.

## 4. Multi-Target Compounds to Treat Schizophrenia

### 4.1. Marketed Drugs—Second and Third Generation Antipsychotics

The second generation antipsychotics, which are nowadays the treatment of choice in cases of schizophrenia and also bipolar disorder, are essentially multi-target compounds. It should be emphasized, however, that many first generation antipsychotics have a complex pharmacological profile, including haloperidol, fluphenazine and even chlorpromazine, as seen in Table 2 [88].

**Figure 2:**
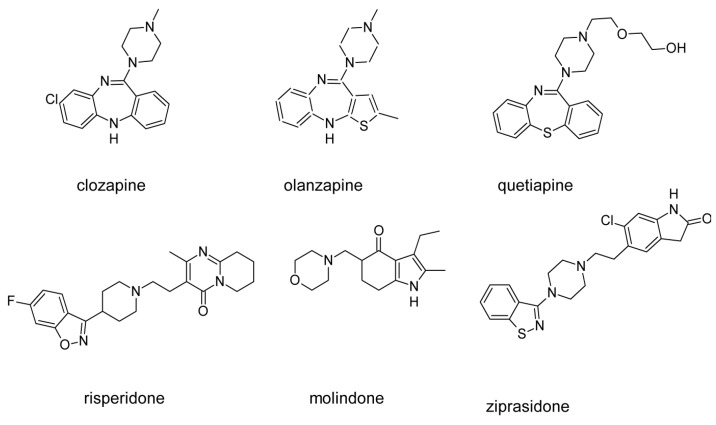
Examples of marketed multi-target second generation antipsychotics [1].

**Figure 3:**
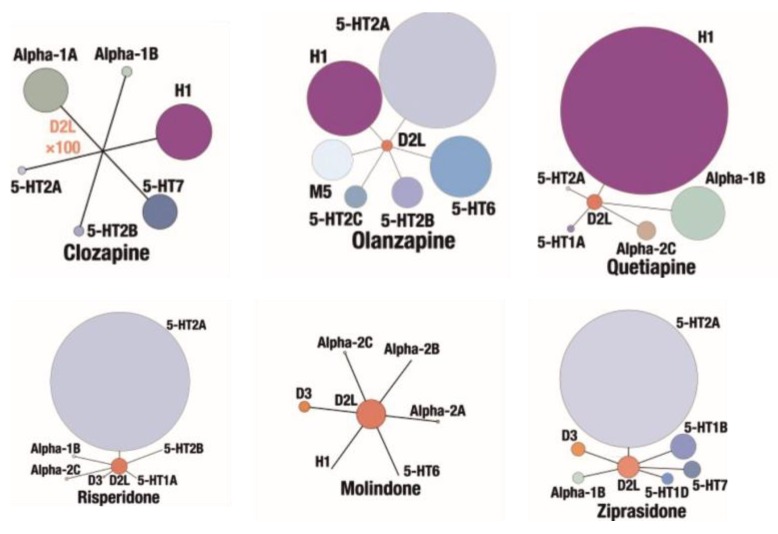
Pharmacological profiles of commonly used second generation antipsychotics, elaborated on the basis of [90] with modifications.

Clozapine (**1**), Figure 2, is a classic example of a “dirty” drug which can be still considered a “gold standard” atypical antipsychotic due to absence of extrapyramidal syndrome (EPS), superiority in treatment of drug resistant schizophrenia and reducing suicidality [88]. Clozapine exerts severe side effects, in particular potentially life-threatening agranulocytosis, but also weight gain, diabetes, and seizures [89]. Both the effectiveness and side effects of clozapine result from its complex pharmacological profile, involving high affinity to many serotonin, dopamine, muscarinic, adrenergic, and other aminergic receptors, as seen in Figure 3 [90].

Some problems with side-effects of clozapine were solved with the introduction of another second generation antipsychotic, olanzapine (**2**), Figure 2. Olanzapine does not cause agranulocytosis but still has metabolic side effects leading to possible weight gain [91] which can be associated with histamine H_1_ receptor signaling [92] and/or the −759C/T and −697G/C polymorphisms of the 5-HT_2C_ receptor gene [93]. Importantly, the side-effect profile of olanzapine can be considered beneficial, with a low incidence of EPS and little increase in prolactin during acute-phase trials [94]. Multi-receptor binding profile of olanzapine [95] involves a nanomolar affinity for dopaminergic, serotonergic, α_1_ adrenergic, and muscarinic receptors, as seen in Figure 3. Olanzapine is also used to treat bipolar disorder.

Similarly, quetiapine (**3**), Figure 2, belongs to atypical antipsychotics, which, besides schizophrenia, are applied to treat bipolar disorder and major depressive disorder. Quetiapine is dopamine D_1_, dopamine D_2_ and serotonin 5-HT_2_ receptor ligand, as seen in Figure 3. Antagonism to α_1_ adrenergic and histamine H_1_ receptor results in side effects like sedation and orthostatic hypotension. Moreover, there are reports about quetiapine misuse and abuse which can be linked with its high affinity for the H_1_ receptor, as antihistamines agents cause rewarding action, compare Figure 3 [96].

Risperidone (**4**), Figure 2 was marketed as the first “non-clozapine” atypical antipsychotic and it is also used to treat the acute manic phase of bipolar disorder. Risperidone is a benzisoxazole derivative with nanomolar affinity for serotonin (5-HT_2A_ and 5-HT_7_) and dopamine D_2_ receptors (its affinity for D_3_ and D_4_ receptors is three times lower), Figure 3 with a 5-HT_2A_/D_2_ affinity ratio of about 20 [11]. It also has a strong affinity for adrenergic (α_1_ and α_2_) receptors, and some affinity for histamine (H_1_) receptors [11]. Pharmacological effect of risperidone is mainly a consequence of antagonism at D_2_ and 5-HT_2A_ receptors, as seen in Figure 3. Its multi-receptor profile resembles this of olanzapine, however risperidone causes sedation less frequent and orthostatic hypotension more often than olanzapine. There are also reports that this drug can increase the level of prolactin and cause arrhythmia.

Molindone (**5**), seen in Figure 2 is a dihydroindolone neuroleptic with dopamine D_2_, D_3_ and D_5_ receptor antagonist activity and affects mainly dopaminergic neurotransmission in the CNS as seen in Figure 3. It is the second generation antipsychotic with atypical pharmacological profile. Its side effects rarely involve sedation and autonomic side effects but more often extrapyramidal side effects (more frequently than other new antipsychotics, although still less frequently than classical drugs). The application of molindone, in contrast to other atypical antipsychotics, does not usually lead to weight gain. Some patients with poor tolerance or response to other drugs can benefit from the treatment with molindone [97].

An example of modern second generation multi-target drug is ziprasidone (**6**), as shown in Figure 2. This antipsychotic is an optimized hybrid of dopamine receptor ligand (D_2_ receptor agonist) and a lipophilic serotonin receptor ligand in which the D_2_ agonist activity is transformed to D_2_ receptor antagonist activity. It also exhibits desirable D_2_/5-HT_2_ ratio of 11 comparable to clozapine, as seen in Figure 3, and has lesser propensity of orthostatic hypotension. Moreover, ziprasidone has been reported not to cause significant weight gain and even to enable some weight loss in obese patients [98].

**Figure 4:**
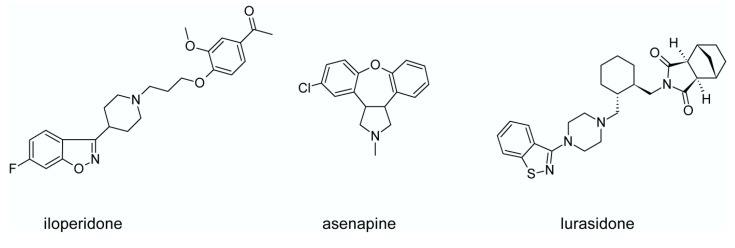
New second generation antipsychotics.

**Figure 5:**
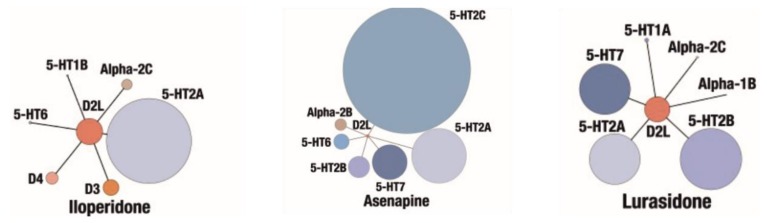
Pharmacological profiles of some new second generation antipsychotics, elaborated on the basis of [90] with modifications.

Some new second generation antipsychotics involve iloperidone (**7**), asenapine (**8**) and lurasidone (**9**), shown in Figure 4, however they have not gained popularity in clinical practice yet. Their pharmacological profiles are presented in Figure 5 [90]. From those three drugs lurasidone seems to be most important. Lurasidone has high antagonist activity at serotonin 5-HT_2A_ and 5-HT_7_ receptors and weaker antagonism at dopamine D_2_ receptor [99]. It has also partial agonist activity at serotonin 5-HT_1A_ receptor, considerable affinity to adrenergic α_2A_ and weaker affinity to muscarinic receptors [99]. Lurasidone is used for treatment of schizophrenia acute bipolar depression. It has low probability of side effects typical for second generation antipsychotics, but higher risk of akathisia in comparison to other atypicals [99].

**Figure 6:**
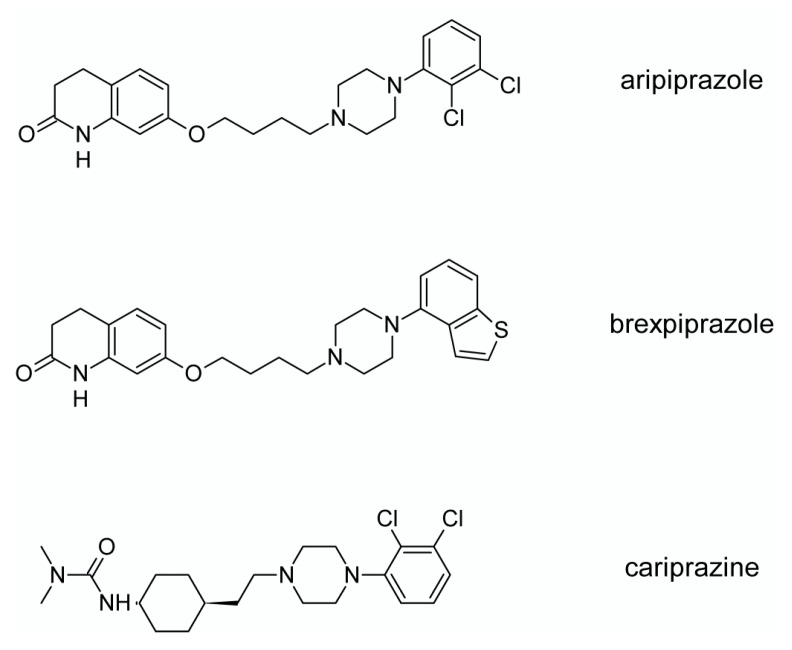
The third generation antipsychotics [1].

**Figure 7:**
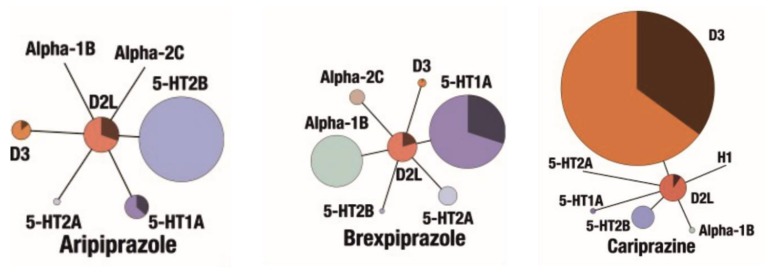
Pharmacological profiles of the third generation antipsychotics, elaborated on the basis of [90] with modifications.

Third generation antipsychotics include aripiprazole (**10**), brexpiprazole (**11**) and cariprazine (**12**), as seen in Figure 6. The mechanism of action of these drugs is still mainly linked to the dopaminergic neurotransmission, shown in Figure 7, however, not to dopamine receptor antagonism but to partial or biased agonism (functional selectivity) [100,101]. Due to partial agonism properties aripiprazole is termed as “dopamine stabilizer” [102,103,104]. Aripiprazole was one of the first functionally selective D_2_ receptor ligands identified that may stabilize the dopaminergic signaling through D_2_ receptor. Although aripiprazole was first described as a partial D_2_ receptor agonist, it was later demonstrated that aripiprazole could behave as a full agonist, a partial agonist, or an antagonist at D_2_ receptor depending upon the signaling readout and cell type interrogated [105]. Aripiprazole is a partial agonist for inhibition of cyclic adenosine monophosphate (cAMP) accumulation through the D_2_ receptor (i.e., Gα signaling) [106,107,108]. In contrast, it has also been reported that aripiprazole is an antagonist in GTPγS binding assays with the D_2_ receptor [107,109]. It was also revealed that aripiprazole failed to activate outward potassium currents following activation of the D_2_ receptor in MES-23.5 cells, indirectly suggesting that it was inactive or possibly an antagonist for Gβγ signaling through the D_2_ receptor [107]. Aripiprazole was also reported to be either an antagonist [110] or a partial agonist [111] for β-arrestin-2 recruitment.

Aripiprazole is also a partial agonist of 5-HT_1A_ and 5-HT_2A_ receptors (much weaker in the latter case) which results in functional antagonism at these receptors, as seen in Figure 7 [90]. In contrast to classical atypical drugs, aripiprazole has higher affinity for dopamine D_2_ receptor than for serotonin 5-HT_2A_ receptor. Clinical application of aripiprazole includes also bipolar disorder, major depression, obsessive-compulsive disorder, and autism. Aripiprazole is characterized by efficacy similar to that of both typical and atypical antipsychotic drugs (except olanzapine and amisulpride) [112]. Aripiprazole resulted in considerably lower weight gain and lower changes in glucose and cholesterol levels in comparison to clozapine, risperidone, and olanzapine [112]. Moreover, aripiprazole led to weaker EPS, less use of antiparkinsonian drugs, and akathisia, in comparison to typical antipsychotic drugs and risperidone [112]. Furthermore, aripiprazole is characterized by better tolerability compared to other antipsychotics [113]. Adverse effects of aripiprazole may include agitation, insomnia, anxiety, headache, constipation or nausea [103].

Brexpiprazole was approved by FDA in 2015 and is a partial agonist of dopamine D_2_, D_3_ and serotonin 5-HT_1A_ receptors, as well as antagonist of 5-HT_2A_, 5-HT_2B_ and 5-HT_7_ receptors, as seen in Figure 7 [114]. Its pharmacological properties are close to those of aripiprazole. In comparison to aripiprazole, brexpiprazole is more potent at 5-HT_1A_ receptors and has less intrinsic activity at D_2_ receptors [115]. Brexpiprazole is applied for treatment of schizophrenia and as an adjunct in major depressive disorder. The adverse effects of this drug invole akathisisa, weight gain, infections of upper respiratory tract, somnolence, headache, and nasopharyngitis.

Approval of both cariprazine and brexpiprazole was in 2015. Cariprazine is a new antipsychotic displaying unique pharmacodynamic and pharmacokinetic properties [116]. As aripiprazole and brexpiprazole, cariprazine is the dopamine D_2_, D_3_ and serotonin 5-HT_1A_ receptors partial agonist, as seen in Figure 7. However, its affinity for dopamine D_3_ receptor is approximately ten times higher than for D_2_ receptors. It is metabolized to two equipotent metabolites, desmethyl cariprazine and didesmethyl cariprazine, of which didesmethyl cariprazine has a half-life of 1 to 3 weeks [116]. Available reports indicate that cariprazine is efficient in management of cognitive and negative symptoms of schizophrenia. It also seems to have antimanic properties and it has a potential to treat bipolar depression [117]. However, currently it is not possible to evaluate antipsychotic potential of cariprazine in comparison to other antipsychotics. Cariprazine may be associated with adverse effects such as sedation, akathisia, weight gain, nausea, constipation, anxiety, dizziness [117].

The problem with the third generation antipsychotics is that they deteriorate the patient’s condition in some patients suffering from schizophrenia. Thus, multi-target second generation antipsychotics are nowadays a gold standard in the schizophrenia treatment, although some patients respond better to the first generation treatment.

### 4.2. Other Multi-Target Compounds for the Treatment of Schizophrenia

Although recently implemented antipsychotics (e.g., cariprazine and brexpiprazole) are the third generation drugs, attempts are still made to design new multi-target ligands, which can be developed into second generation antipsychotics or better third generation drugs. These efforts will be presented in this chapter.

#### 4.2.1. Modifications of Marketed Drugs

**Figure 8:**
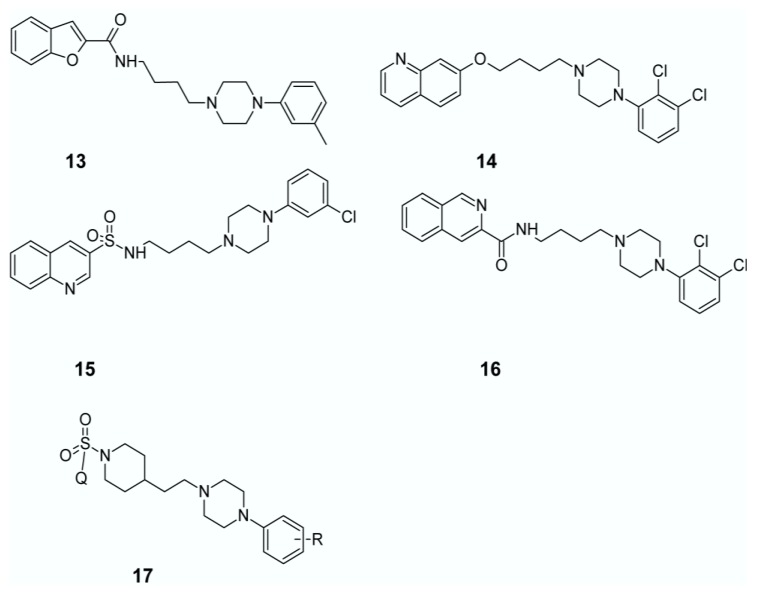
Novel potential multi-target antipsychotics derived from aripiprazole structure. Q: quinolone or isoquinoline.

In recent years, a number of research groups studied halogenated arylpiperazines as a privileged scaffold active in CNS resulting in antipsychotics such as aripiprazole, trazodone and cariprazine [118]. The multimodal receptor profile of aripiprazole (5-HT_1A_, 5-HT_2A_, 5-HT_7_, D_2_ and D_3_ receptors), as well as its functional profile as a partial agonist of D_2_ and 5-HT_1A_ receptors and antagonist of 5-HT_2A_ and 5-HT_7_ sites, makes it a good starting point to design compounds with antipsychotic, antidepressant, and anxiolytic activity [119]. Expanding the concept of mixed serotonin/dopamine receptor agonists as novel antipsychotics, Butini et al. designed a series of aripiprazole analogs that combined high affinity for 5-HT_1A_ and 5-HT_2A_ receptors, low affinity for D_2_ receptors and high affinity for D_3_ receptors. The structures of the compounds were based mainly on the 2,3-dichlorophenylpiperazine core structure, which was functionalized with isoquinoline-amide and quinolone- and isoquinoline-ether moieties, e.g., compound (**13**), compared in Figure 8. The study revealed that the optimal serotonin/dopamine receptor affinity balance was characterized by compounds with isoquinoline or benzofurane rings as heteroatomic systems [120]. As a continuation of their studies they developed a series bishetero(homo)arylpiperazines as novel and potent multifunctional ligands characterized by high affinity to D_3_, 5-HT_1A_ and low occupancy at D_2_ and 5-HT_2C_ receptors [121].

In 2013 Zajdel et al. developed a series of new quinoline- and isoquinoline-sulfonamide analogs of aripiprazole to explore the effect of the replacement of the ether/amide moiety with sulfonamide, as well as the localization of a sulfonamide group in the azine moiety, (**14**–**16**), see in Figure 8. In this study, two specific compounds displayed 5-HT_1A_ agonistic, D_2_ partial agonistic and 5-HT_2A_/5-HT_7_ antagonistic activity, thus resulting in significant antidepressant activity in mice models of depression [119]. Furthermore, the 4-isoquinolinyl analog (*N*-(4-(4-(2,3-dichlorophenyl)piperazin-1-yl)butyl)isoquinoline-4-sulfonamide) not only exhibited a similar receptor binding and functional profile but also displayed significant antipsychotic activity in MK-801-induced hyperlocomotor activity in mice [119]. These results supported the study previously conducted by Zajdel and coworkers in 2012, which reported on quinoline- and isoquinoline-sulfonamide derivatives of long-chain arylpiperazines with 3- or 4-chloro-phenylpiperazine moieties as potential antidepressant, anxiolytic and antipsychotic agents [122].

Partyka et al. inspired by previous findings on a group of *N*-alkylated azinesulfonamides, synthesized a series of 15 azinesulfonamides of phenylpiperazine derivatives, based on 4-(4-{2-[4-(4-chlorophenyl)-piperazin-1-yl]-ethyl}-piperidine-1-sulfonyl)-isoquinoline with semi-rigid alkylene spacer (**17**), as seen in Figure 8, and evaluated them as multimodal dopamine/serotonin receptor ligands. The study allowed to identify compound 5-({4-(2-[4-(2,3-dichlorophenyl)piperazin-1-yl]ethyl)piperidin-1-yl}sulfonyl)quinolone which behaved as mixed D_2_/5-HT_1A_/5-HT_7_ receptor antagonist. Preliminary pharmacological in vivo evaluation showed that compound was active in MK-801-evoked hyperactivity test in mice, and produced antidepressant-like activity in a mouse model of depression. Further studies in the area of CNS agents with multiple mode of action might confirmed its broad-based efficacy in the treatment of comorbid symptoms of schizophrenia/depression/anxiety [123].

**Figure 9:**
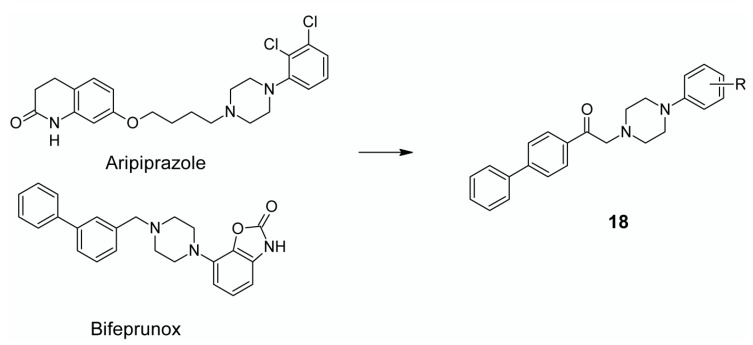
Design of multi-target hybrid compound based on aripiprazole and bifeprunox scaffold.

In 2007 the atypical antipsychotic bifeprunox [1-(2-oxo-benzoxazolin-7-yl)-4-(3-biphenyl) methylpiperazine], with dual D_2_ and 5-HT_1A_ partial agonist activity, was filed for regulatory approval with the Food and Drug Administration (FDA), however the application was rejected owing to the weakness of evidence submitted and the death of a patient involved in the clinical trials. Nevertheless, through various molecular modification studies, it was established that the phenylpiperazine moiety is responsible for its antiserotonergic and antidopaminergic activity of this compound [120]. Based on these findings and the anti-inflammatory, nitric oxide synthase inhibitory activity, antidiabetic and antifungicidal activity of biphenyl compounds, a hybrid structure comprising a biphenyl and arylpiperazine moiety with an acetyl linker was designed [124]. In this study Bhosale et al. focused on combining the beneficial effects of the biphenyl moiety of bifeprunox with the methylpiperazine moiety of the aripiprazole. The newly designed hybrid antipsychotic scaffold (**18**) is presented in Figure 9.

#### 4.2.2. Other Multi-Target Compounds with Potential Application for the Treatment of Schizophrenia

**Figure 10:**
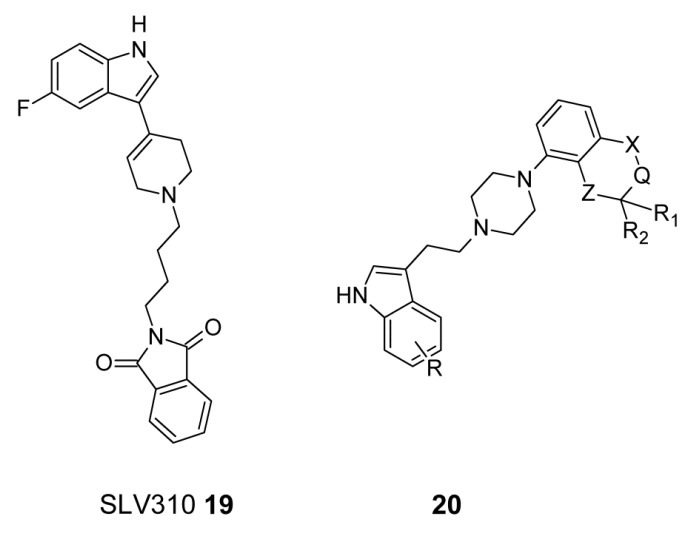
Dopamine D_2_ receptor antagonists with selective serotonin reuptake inhibitor (SSRI) activity.

It has been reported that the adjunctive usage of a neuroleptic together with selective serotonin reuptake inhibitor (SSRI), e.g., fluvoxamine, fluoxetine or citalopram is beneficial for the treatment of negative symptoms of schizophrenia without increasing EPS [125]. In this regard van Hes et al. elaborated SLV310, seen in Figure 10, (**19**), as a novel, potential antipsychotic displaying the interesting combination of potent dopamine D_2_ receptor antagonism and serotonin reuptake receptor inhibition in one molecule which can be useful in treatment a broad range of symptoms in schizophrenia [126]. Subsequently the same research group obtained a series of compounds displaying D_2_ receptor antagonism as well as SSRI properties by connecting the aryl piperazine of a neuroleptic with the indole moiety of a SSRI through alkyl chain in order to obtain promising antipsychotic agents, seen in Figure 10, (**20**). Optimization of length of the alkyl linker chain, substitution pattern of the indole moiety and bicyclic heteroaryl part has led to the maximally potent compound. Further, the molecular modelling studies have shown that the bifunctional activity of compound can be explained by its ability to adopt two different conformations fitting either D_2_ receptor or SR pharmacophore without the disadvantages of potential pharmacokinetic interactions [127].

**Figure 11:**
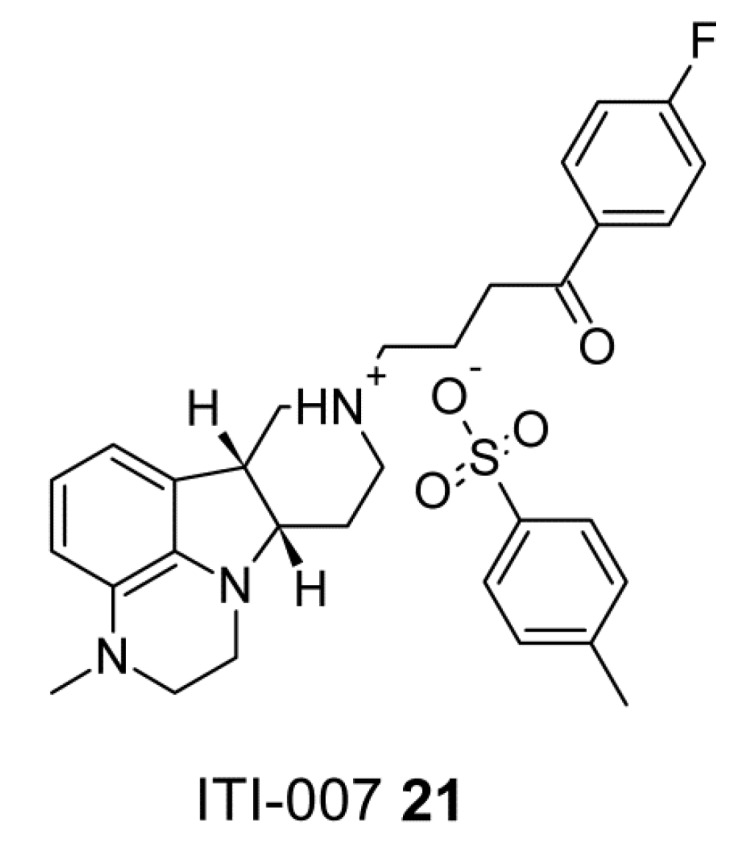
ITI-007, a potent 5-HT_2A_ receptor antagonist, postsynaptic D_2_ receptor antagonist and inhibitor of serotonin transporter.

Li et al. reported synthesis and structure-activity relationships of a series of tetracyclic butyrophenones that display high affinities to serotonin 5-HT_2A_ and dopamine D_2_ receptors [128]. In particular, ITI-007 (4-((6bR,10aS)-3-methyl-2,3,6b,9,10,10a-hexahydro-1H,7H-pyrido[3′,4′:4,5]pyrrolo[1,2,3-de]quinoxalin-8-yl)-1-(4-fluorophenyl)-butan-1-one 4-methylbenzenesulfonate), seen in Figure 11, (**21**), was found to be a potent 5-HT_2A_ receptor antagonist, postsynaptic D_2_ receptor antagonist and inhibitor of serotonin transporter [128].

**Figure 12:**
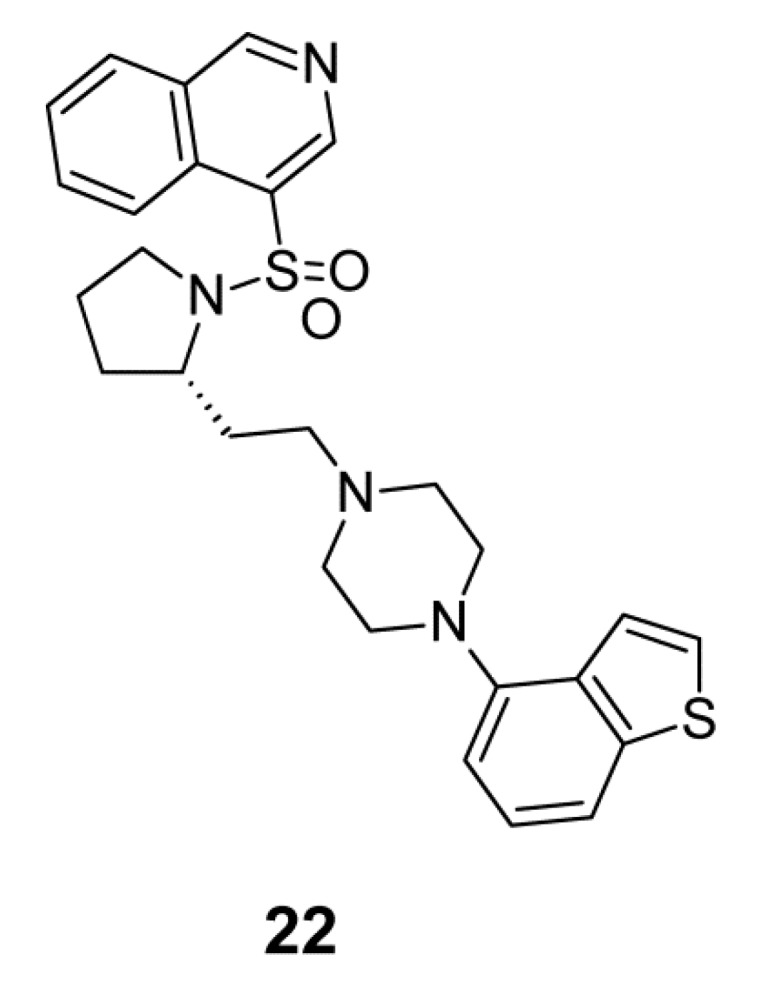
Multi-target ligand of aminergic G protein-coupled receptors (GPCRs) with SERT inhibitory properties.

In the latest study, Zajdel et al. [129] designed, synthesized and characterized a new series of azinesulfonamides of alicyclic amine derivatives with arylpiperazine/piperidine scaffold. Structure-activity studies of this compound series disclosed that the (isoquinolin-4-ylsulfonyl)-(*S*)-pyrrolidinyl fragment and the 1,2-benzothiazol-3-yl- and benzothiophen-4-yl-piperazine fragments were beneficial for affinity to 5-HT_1A_, 5-HT_2A_, 5-HT_6_, 5-HT_7_, D_2_ and D_3_ receptors. Furthermore, binding of these compounds with 5-HT_6_ receptor depended on the stereochemistry of the alicyclic amine. Within this compound series, (*S*)-4-((2-(2-(4-(benzo[b]thiophen-4-yl)piperazin-1-yl)ethyl) pyrrolidin-1-yl) sulfonyl) isoquinoline, seen in Figure 12, (**22**), was identified as a potential novel antipsychotic. This compound is also characterized by blockade to SERT. Because it reverses PCP-induced hyperactivity and avoidance behavior in the CAR test, (**22**) it can be used to treat positive symptoms of schizophrenia. Next, its ability to reverse the social interaction deficit in a ketamine model and memory impairment in phencyclidine (PCP)- and ketamine-disrupted conditions reveals that that drug can improve the negative symptoms and has procognitive activity. Importantly, this compound did not have cardiac toxicity and tendency of inducing catalepsy [129].

**Figure 13:**
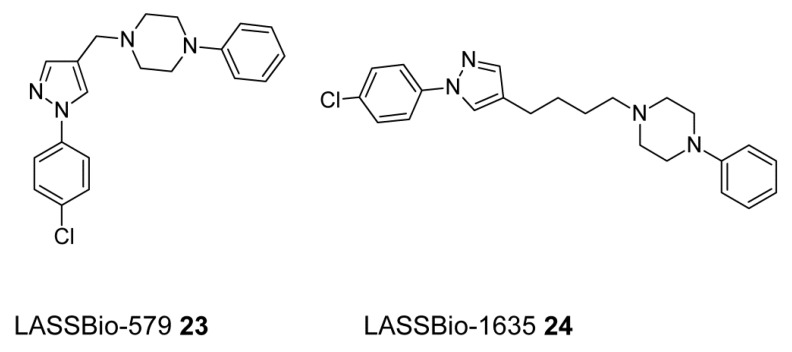
Potential multi-target (dopamine and serotonin receptor ligands) antipsychotics.

In order to obtain novel antipsychotics Menegatti et al. designed and synthesized a series of N-phenylpiperazine derivatives [130]. A few compounds, i.e., 1-[1-(4-chlorophenyl)-1H-pyrazol-4-ylmethyl]-4-phenyl-piperazine (LASSBio-579, **23**, Figure 13), 1-phenyl-4-(1-phenyl-1H-[1,2,3]triazol-4-ylmethyl)-piperazine (LASSBio-580) and 1-[1-(4-chlorophenyl)-1H-[1,2,3]triazol-4-ylmethyl]-4-phenyl-piperazine (LASSBio-581) were selected based on potential antipsychotic activity. It was found that LASSBio-579 is the most promising of the three compounds, thanks to its affinity to both dopamine and serotonin receptors, in particular agonist activity at 5-HT_1A_ receptor [131]. Thus, this multi-target compound was active in animal models of psychosis and reversed the catalepsy induced by WAY 100,635, Furthermore, co-administration of sub-effective doses of LASSBio-579 with sub-effective doses of clozapine or haloperidol prevented apomorphine-induced climbing without induction of catalepsy [131].

In 2013, another team synthesized and made a pharmacological evaluation of the antipsychotic homologues of the lead compound LASSBio-579. The applied homologation approach turned out to be appropriate for increasing the affinity of these compounds to the 5-HT_2A_ receptors, with no significant changes in the affinity for the D_2_, D_4_ and 5-HT_1A_ receptors. In this context, (1-(4-(1-(4-chlorophenyl)-1*H*-pyrazol-4-yl) butyl)-4-phenylpiperazine) (LASSBio-1635, **24**), Figure 13 was the most promising derivative with a ten-fold higher affinity for the 5-HT_2A_ receptor than its parent compound. Moreover, LASSBio-1635 displayed beneficial antagonistic efficacy at the 5-HT_2A_ receptors. Next, LASSBio-1635 has also a 4-fold higher affinity for α_2_ adrenergic receptors in comparison to LASSBio-579 and the favorable antagonistic efficacy. This multi-target ligand fully prevented the apomorphine-induced climbing in mice and prevented the ketamine-induced hyperlocomotion at doses with no effect on the mice locomotor activity [132].

**Figure 14:**
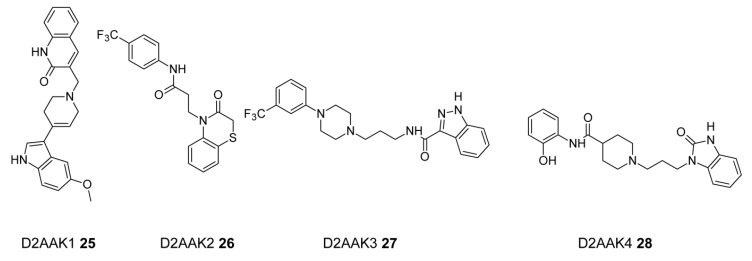
Multi-target compounds obtained in structure-based virtual screening.

In order to search for potential multi-target antipsychotics, Kaczor et al. [86] performed structure-based virtual screening using a D_2_ receptor homology model in complex with olanzapine or chlorprothixene. As a result of a screen they selected 21 compounds, which were subjected to experimental validation. From 21 compounds tested, they found ten D_2_ ligands (47.6% success rate, among them D_2_ receptor antagonists as expected) possessing additional affinity to other receptors tested, in particular to 5-HT_1A_ (partial agonists) and 5-HT_2A_ receptors (antagonists). The affinity of the compounds ranged from 58 nM to about 24 µM. Similarity and fragmental analysis indicated a significant structural novelty of the identified compounds. The best compound (D2AAK1, **25**) has affinity of 58 nM to D_2_ receptor and nanomolar or low micromolar affinity to D_1_, D_3_, 5-HT_1A_ and 5-HT_2A_ receptors. D2AAK1 is an antagonist at D_2_ receptor and 5-HT_2A_ receptor and a partial agonist at 5-HT_1A_ receptor which is favorable for antipsychotic activity [131]. They found one D_2_ receptor antagonist (D2AAK2, **26**) that did not have a protonatable nitrogen atom which is a key structural element of the classical D_2_ pharmacophore model necessary to interact with the conserved Asp(3.32). This compound exhibited over 20-fold binding selectivity for the D_2_ receptor compared to the D_3_ receptor. The four best compounds (D2AAK1–D2AAK4, **25**–**28**, Figure 14) were subjected to in vivo evaluation. In particular compound D2AAK1 decreased amphetamine-induced hyperactivity (when compared to the amphetamine-treated group), measured as spontaneous locomotor activity in mice. In addition, in a passive avoidance test this compound improved memory consolidation after acute treatment in mice. Elevated plus maze tests indicated that D2AAK1 compound induced anxiogenic activity 30 min after acute treatment and anxiolytic activity 60 min after administration [133].

AVN-101 (**29**, Figure 12) is another multi-target drug candidate that has an advantageous target fingerprint of activities with prevalent affinity to serotonin receptors, mainly 5-HT_7_, 5-HT_6_, 5-HT_2A_, and 5-HT_2C_, as well as to adrenergic α_2B_, α_2A_, and α_2C_ and histamine H_1_ and H_2_ receptors. The AVN-101 exhibits positive effects in the animal models of both impaired and innate cognition. It also exhibited significant anxiolytic and anti-depressant capabilities [134].

**Figure 15:**
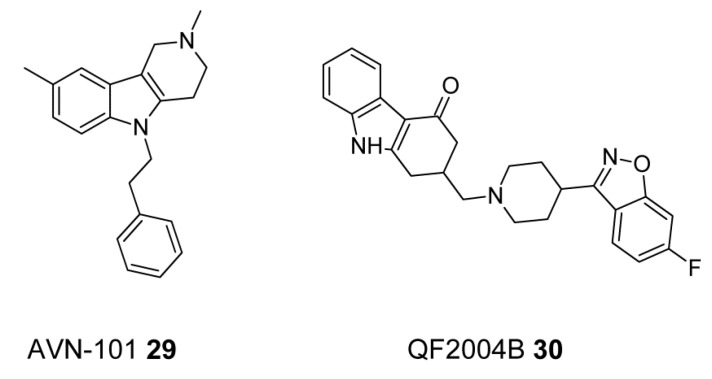
Multi-target ligands of aminergic GPCRs as potential antipsychotics.

2-[4-(6-fluorobenzisoxazol-3-yl)piperidinyl]methyl-1,2,3,4-tetrahydro-carbazol-4-one (QF2004B), a conformationally constrained butyrophenone analog (**30**, Figure 15) has a multi-receptor profile with affinities similar to those of clozapine for serotonin (5-HT_2A_, 5-HT_1A_, and 5-HT_2C_), dopamine (D_1_, D_2_, D_3_ and D_4_), alpha-adrenergic (α_1_, α_2_), muscarinic (M_1_, M_2_) and histamine H_1_ receptors. In addition, QF2004B mirrored the antipsychotic activity and atypical profile of clozapine in a broad battery of in vivo tests including locomotor activity, apomorphine-induced stereotypies, catalepsy, apomorphine- and DOI (2,5-dimethoxy-4-iodoamphetamine)-induced prepulse inhibition (PPI) tests. These results point to QF2004B as a new lead compound with a relevant multi-receptor interaction profile for the discovery and development of new antipsychotics [135].

**Figure 16:**
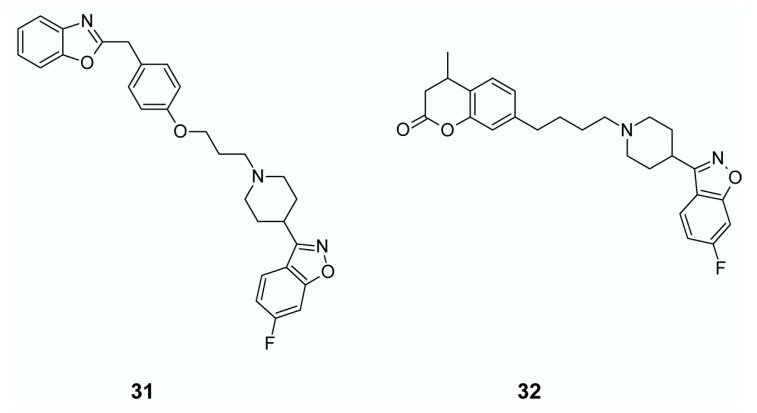
Multi-target ligands of aminergic GPCRs as potential antipsychotics with low affinity to off-targets.

Searching for potential multi-target antipsychotics, Huang et al. [136] obtained a series of compounds bearing benzoxazole-piperidine (piperazine) scaffold with considerable dopamine D_2_ and serotonin 5-HT_1A_ and 5-HT_2A_ receptor binding affinities. The best compound (**31**, Figure 16) had high affinity to D_2_, 5-HT_1A_ and 5-HT_2A_ receptors, but low affinities foroff-targets (the 5-HT_2C_ and histamine H_1_ receptors and human ether-a-go-go-related gene (hERG) channels). This compound diminished apomorphine-induced climbing and DOI-induced head twitching without observable catalepsy, even at the highest dose tested making it a promising candidate for multi-target antipsychotic treatment.

Chen et al. [137] obtained potential antipsychotic coumarin derivatives, having potent dopamine D_2_, D_3_, and serotonin 5-HT_1A_ and 5-HT_2A_ receptor affinities. The best compound, seen in 32, Figure 16, also possesses low affinity for 5-HT_2C_ and H_1_ receptors and hERG channels. In behavioral studies this compound inhibited apomorphine-induced climbing behavior, MK-801-induced hyperactivity, and the conditioned avoidance response without observable catalepsy. Further, fewer preclinical side effects were observed for (**32**) in comparison to risperidone in assays that measured prolactin secretion and weight gain.

**Figure 17:**
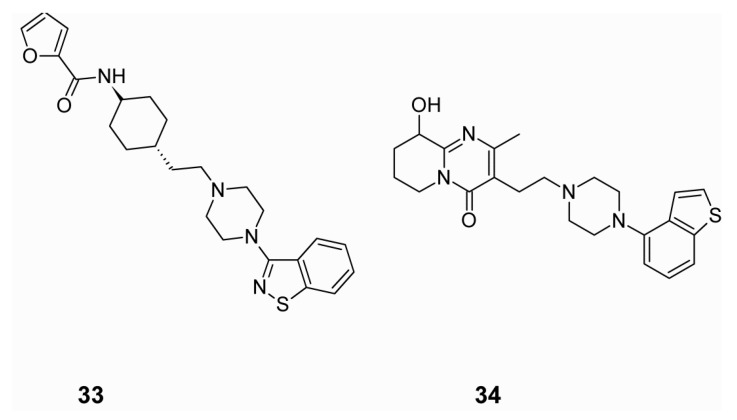
Potential multi-target antipsychotics with low probability of adverse effects.

Another group synthesized a series of benzisothiazolylpiperazine derivatives combining potent dopamine D_2_ and D_3_, and serotonin 5-HT_1A_ and 5-HT_2A_ receptor affinities [138]. The best compound, as seen in (**33**), Figure 17, had significant affinity for D_2_, D_3_, 5-HT_1A_, and 5-HT_2A_ receptors, accompanied by a 20-fold selectivity for the D_3_ versus D_2_ subtype, and a low affinity for muscarinic M_1_ and for hERG channels. In animal studies this compound blocked the locomotor-stimulating effects of phencyclidine, inhibited conditioned avoidance response, and improved the cognitive impairment in the novel object recognition tests in rats [138].

In a recent study Xiamuxi et al. [139] reported a series of tetrahydropyridopyrimidinone derivatives, possessing potent dopamine D_2_, serotonin 5-HT_1A_ and 5-HT_2A_ receptors affinities. The most promising compound, seen in (**34**), Figure 17, displayed high affinity to D_2_, 5-HT_1A_, and 5-HT_2A_ receptors, with low affinity to α_1A_, 5-HT_2C_, H_1_ receptors and hERG channels. In animal models, this compound diminished phencyclidine-induced hyperactivity with a high threshold for catalepsy induction.

**Figure 18:**
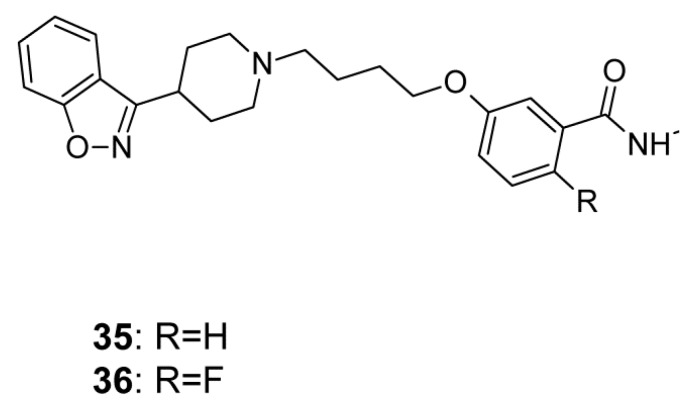
Benzamides as potential multi-target antipsychotics with low probability of side effects.

In another new study Yang et al. [140] designed a series of benzamides, with potent dopamine D_2_, serotonin 5-HT_1A_ and 5-HT_2A_ receptor affinity. Two best compounds, seen in (**35**) and (**36**), Figure 18, were not only potent D_2_, 5-HT_1A_, and 5-HT_2A_ receptor ligands, but they were weak binders of 5-HT_2C_, H_1_ receptors and hERG channels. In behavioral studies these compounds decreased phencyclidine-induced hyperactivity with a high threshold for catalepsy induction.

## 5. Conclusions and Perspectives

The growing pace of life promotes mental disorders. Pharmacotherapy for schizophrenia is nowadays very effective, in particular regarding treating positive symptoms of the disease, but at the same time there is a tremendous, unmet clinical need for the therapy of negative and cognitive symptoms, as well as for the management of drug resistant schizophrenia. Over the last half century, there has been only limited progress in the innovating mechanisms of action and the developing novel therapeutic agents for the treatment of schizophrenia. However, the breadth of potential goals and tested compounds clearly shows interest and importance in the pursuit of innovative drug development. A multi-target approach to drug design and discovery is now a hot topic in medicinal chemistry, in particular for the treatment of complex diseases such as schizophrenia. It should be emphasized that regarding management of schizophrenia, nothing more effective than multi-target treatment has been proposed. Involvement of nicotinic and glutamatergic targets in modern multi-target drugs can be beneficial for the treatment of negative symptoms and cognitive impairment. Another potential strategy is exploration novel signaling mechanisms concerning in particular GPCRs, such as allosteric modulation, biased signaling (functional selectivity), and receptor oligomerization. However, this approach will also be more promising when it involves multiple targets. In summary, as current multi-target antipsychotics are mainly orthosteric ligands of aminergic GPCRs with SSRI or SERT inhibitory activity in some cases, there is a huge unexplored area to include other receptors and enzymes as drug targets and to explore the wealth of signaling mechanism beyond the ternary complex model of GPCRs.

## Figures and Tables

**Table 1:** Potential clinical benefits and side effects related to the mechanisms of action of antipsychotics [10,11,12].

Mechanism of Action	Clinical Efficacy	Possible Side Effects
D_2_ antagonism	↓Positive symptoms	Extrapyramidal symptoms (EPS) ↓Negative symptoms ↑Cognitive symptoms ↑Drowsiness
D_2_ partial agonism	↓Positive symptoms ↓Negative symptoms ↓Cognitive symptoms	Little or no EPS Behavioral activation
D_3_ antagonism		↑Endocrine dysfunction ↑Weight gain ↑Sexual dysfunction
5-HT_2A_ antagonism	↓Negative symptoms	↓EPS ↓Hyperprolactinemia
5-HT_1A_ partial agonism	↓Negative symptoms ↓Cognitive symptoms ↓Anxiety symptoms ↓Depressive symptoms	↓EPS ↓Hyperprolactinemia
5-HT_2C_ antagonism		↑Weight gain ↑Appetite
M_1_ antagonism	↓EPS	↑Anticholinergic symptoms, e.g., dry mouth, constipation, tachycardia ↑Drowsiness ↑Cognitive impairment
M_1_ agonism	↓Psychotic symptoms ↓Cognitive symptoms	
M_3_ antagonism		↑Type 2 diabetes mellitus ↑Hyperglycemic hyperosmolar syndrome ↑Diabetic ketoacidosis
H_1_ antagonism		↑Weight gain ↑Drowsiness ↑Hypotension
α_1_-antagonism		↑Dizziness ↑Drowsiness ↑Tachycardia ↓Blood pressure ↑Orthostatic hypotension
α_2_-antagonism	↓Depressive symptoms	↑Anxiety ↑Tachycardia ↑Tremor ↑Dilated pupils ↑Sweating
β-antagonism		↑Orthostatic hypotension ↑Sedation ↑Sexual dysfunction
Glutamate modulation	↓Positive symptoms ↓Negative symptoms ↓Cognitive symptoms ↓Illness progression	

Legend: ↓ Decreasing ↑ Increasing.

**Table 2:** Relative neurotransmitter receptor affinities for first, second and third generation antipsychotics and involved side effects.

Drugs Generation	Examples	Receptors								Potential Side Effect
		D_1_	D_2_	D_3_	D_4_	5-HT_2A_	α_1_	H_1_	M_1_	
First	Chloropromazine	+	++	+++	+	+++	++	++	++	extrapyramidal symptoms such as dyskinesia, dystonias, akathisia, unwanted movements, muscle breakdown, tremors, rigidity and elevated prolactin
	Haloperidol	+	+++	+	+	0	+	0	0	
	Benperidol	0	+++	++		++	+	0	0	
	Fluspirilene	+	+++	+++		+	0	0	0	
	Thioridazine	+	++	++	+	++	++	+	+	
Second	Clozapine	++	+	++	+++	++	+		+++	hypotension, tachycardia, agranulocytosis
	Olanzapine	++	+++	+	++	+++	++	++	++	sedation, weight gain
	Risperidone	+	++	+	+	+++	++	+++	0	orthostatic hypotension, insomnia, restlessness, anxiety, headaches, agitation, extrapyramidal symptoms (EPS), rhinitis, sedation, fatigue, ocular disturbances, dizziness, palpitations, weight gain, diminished sexual desire, erectile and ejaculatory dysfunction, orthostatic dysregulation, reflex tachycardia, gastrointestinal complaints, nausea, rash, galactorrhea and amenorrhea
	Quetiapine	+	++	+++	++	+++	+++	+++	0	drowsiness, dizziness, headache, withdrawal symptoms, increased triglycerides, increased total cholesterol
	Ziprasidone	++	+++	+++	+++	+++	++	+	0	parkinsonism, headaches, rhinitis, orthostatic hypotension, tachykardia
Third	Aripiprazole	0	+++	++	+	+++	+	+	0	hyperglycaemia, headache, extrapyramidal symptoms

+++ = high, ++ = moderate, + = low, 0 = minimal to none.

## Abbreviations

3D QSAR	Three-dimensional structure-activity relationship
AMPA	α-Amino-3-hydroxy-5-methyl-4-isoxazolepropionic acid
cAMP	Cyclic adenosine monophosphate
CAR	Conditioned avoidance response
CNS	Central nervous system
cNT PDEs	Cyclic nucleotide phosphodiestereases
EPS	Extrapyramidal symptoms
FDA	Food and Drug Administration
GABA	γ-Aminobutyric acid
GPCRs	G protein-coupled receptors
GSK-3	Glycogen synthase kinase-3
GTP	Guanosine-5′-triphosphate
LSD	Lysergic acid diethylamide
MTDs	Multi-target drugs
NMDA	*N*-methyl-d-aspartate
PCP	Phencyclidine
SSRI	Selective serotonin reuptake inhibitor
VTA	Ventral tegmental area
